# Identification of germ cell-specific *Mga* variant mRNA that promotes meiosis via impediment of a non-canonical PRC1

**DOI:** 10.1038/s41598-021-89123-5

**Published:** 2021-05-06

**Authors:** Yuka Kitamura, Kousuke Uranishi, Masataka Hirasaki, Masazumi Nishimoto, Ayumu Suzuki, Akihiko Okuda

**Affiliations:** 1 Division of Biomedical Sciences, Research Center for Genomic Medicine, Saitama Medical University, 1397-1, Yamane Hidaka, Saitama, 350-1241 Japan; 2 Department of Clinical Cancer Genomics, International Medical Center, Saitama Medical University, 1397-1, Yamane Hidaka, Saitama, 350-1241 Japan; 3 Biomedical Research Center, Saitama Medical University, 1397-1, Yamane Hidaka, Saitama, 350-1241 Japan

**Keywords:** Computational biology and bioinformatics, Developmental biology, Molecular biology

## Abstract

A non-canonical PRC1 (PRC1.6) prevents precocious meiotic onset. Germ cells alleviate its negative effect by reducing their amount of MAX, a component of PRC1.6, as a prerequisite for their *bona fide* meiosis. Here, we found that germ cells produced *Mga* variant mRNA bearing a premature termination codon (PTC) during meiosis as an additional mechanism to impede the function of PRC1.6. The variant mRNA encodes an anomalous MGA protein that lacks the bHLHZ domain and thus functions as a dominant negative regulator of PRC1.6. Notwithstanding the presence of PTC, the *Mga* variant mRNA are rather stably present in spermatocytes and spermatids due to their intrinsic inefficient background of nonsense-mediated mRNA decay. Thus, our data indicate that meiosis is controlled in a multi-layered manner in which both MAX and MGA, which constitute the core of PRC1.6, are at least used as targets to deteriorate the integrity of the complex to ensure progression of meiosis.

## Introduction

Meiosis is a specialized type of cell division, which converts a cell from diploid to haploid^1^. Stra8 is a crucial positive regulator of meiosis in mammals^2^ and molecular cloning of Meiosin by Ishiguro et al.^3^, which functions by forming a complex with Stra8, significantly advanced our understanding of the molecular bases of Stra8-dependent meiotic onset. In contrast to the STRA8/MEIOSIN complex, polycomb repressive complex 1 (PRC1) is a negative regulator of meiotic onset in mammals^4,5^. The PRC1 family includes six distinct subtypes, PRC1.1–6, which share RING1A or RING1B as a common subunit bearing enzymatic activity for ubiquitination of histone H2A at lysine 119, but differ in their composition of other subunits^6–10^. The PRC1 family is largely classified into two groups, i.e., canonical (PRC1.2 and PRC1.4) and non-canonical (PRC1.1, PRC1.3, PRC1.5, and PRC1.6) complexes. Canonical PRC1s containing chromobox proteins bind to chromatin via an interaction with the histone modification H3K27me3 catalyzed by PRC2. Therefore, recruitment of canonical PRC1s to chromatin occurs after binding of PRC2^11–13^. However, non-canonical PRC1s such as PRC1.6 are recruited to chromatin prior to binding of PRC2 using their distinct ways one another^6,14,15^. For example, recruitment of PRC1.1 to genome target sites is dependent on its KDM2B subunit that recognizes non-methylated CpG islands^16^. In terms of PRC1.6, two DNA-binding proteins of the complex, i.e., MGA and E2F6, are used for its direct binding to genomic sites^6,17^. It is also known that PCGF6, L3MBTL2, RYBP, and YAF2 substantially contribute to recruitment of PRC1.6 to chromatin^18,19^. We and others have recently demonstrated that PRC1.6 acts as a strong blocker of ectopic and precocious onset of meiosis in embryonic stem cells and germ cells, respectively^20–22^, which suggests that a previous report by Yokobayashi et al.^4^ showing strong induction of meiosis by deprivation of RING1B, a common component in the PRC1 family, is largely accounted for by disruption of PRC1.6. We have also demonstrated that germ cells transcriptionally and/or post-translationally reduce their amount of MAX, a component of PRC1.6, to liberate them from PRC1.6-dependent repression at the timing of or immediately prior to meiotic onset^21^. However, because meiosis is very dynamic and central process in gametogenesis, we assumed that inactivation of PRC1.6 in germ cells is not solely dependent on the reduction of Max protein levels, but regulated in a multi-layered manner to ensure meiosis as a safeguarding system.

Here, we addressed this issue by searching for potential exon sequences in genes encoding one of the components of PRC1.6 using SpliceAI, a recently developed deep neural network^23^, which led to identification of *Mga* variant mRNA carrying a novel sequence with a premature termination codon (PTC). The variant mRNA generated by alternative splicing encodes a carboxy-terminally truncated MGA protein that functions as a dominant negative regulator of PRC1.6 owing to the lack of the basic helix-loop-helix/leucine zipper (bHLHZ) domain. We also found that this variant mRNA is specifically present in meiotic germ cells and round spermatids. Furthermore, our data demonstrated that this salient expression profile of *Mga* variant mRNA represented the combined consequence of preferential production of the variant mRNA in germ cells including spermatogonia and inefficient background of PTC-dependent nonsense-mediated mRNA decay (NMD) in specific subpopulations of germ cells, i.e., meiotic germ cells and spermatids.

## Results

### Exon inclusion is the most prevalent type of alternative splicing during transition from mitosis to meiosis in germ cells

We have previously demonstrated that germ cells physiologically reduce their amount of MAX protein that constitutes the core of PRC1.6 with MGA to de-repress meiosis-related genes prior to meiotic onset^21^. In this study, we explored the possibility of an additional molecular mechanism that inactivates the function of PRC1.6 to ensure meiosis. Because the testis is known for its prevalence of alternative splicing similar to the brain^24,25^, we pursued the possibility of involvement of alternative splicing in facilitating meiosis by deteriorating the function of PRC1.6. First, we examined which stages in spermatogenesis and which types of alternative splicing were prevalent in germ cells by inspecting publicly reported RNA sequence data. These analyses revealed that alternative splicing occurred most actively during transition of spermatogonia to preleptotene spermatocytes (Supplementary Fig. S1A). In terms of the types of alternative splicing, the skipping exon (SE) type was the most prevalent, which accounted for more than 50% of all alternative splicing events among the five distinct splicing types. Furthermore, our analyses revealed that gain of a novel exon was approximately twice as frequent as loss of an exon among the SE-type alternative splicing events (Supplementary Fig. S1B). Moreover, our data revealed that most transcripts that gained or lost an exon around transition of spermatogonia to preleptotene spermatocytes maintained their forms by de novo synthesis and/or stabilization at least up to the round spermatid stage (Supplementary Fig. S1C). We also classified alternative splicing events in neural progenitor cells and mesenchymal stem cells using publicly reported RNA sequence data. These analyses revealed that SE was also the most prevalent alternative splicing during differentiation of neural progenitor cells and mesenchymal stem cells (Supplementary Fig. S1A). However, comparisons of genes that gained a new exon revealed that these three gene populations of germ cells, neural cells and mesenchymal stem cells barely overlapped (Supplementary Fig. S1D), which indicated that at least genes subjected to this type of alternative splicing were selected distinctly in each cell type.

### Identification of testis-specific *Mga* variant mRNA

**Figure 1:**
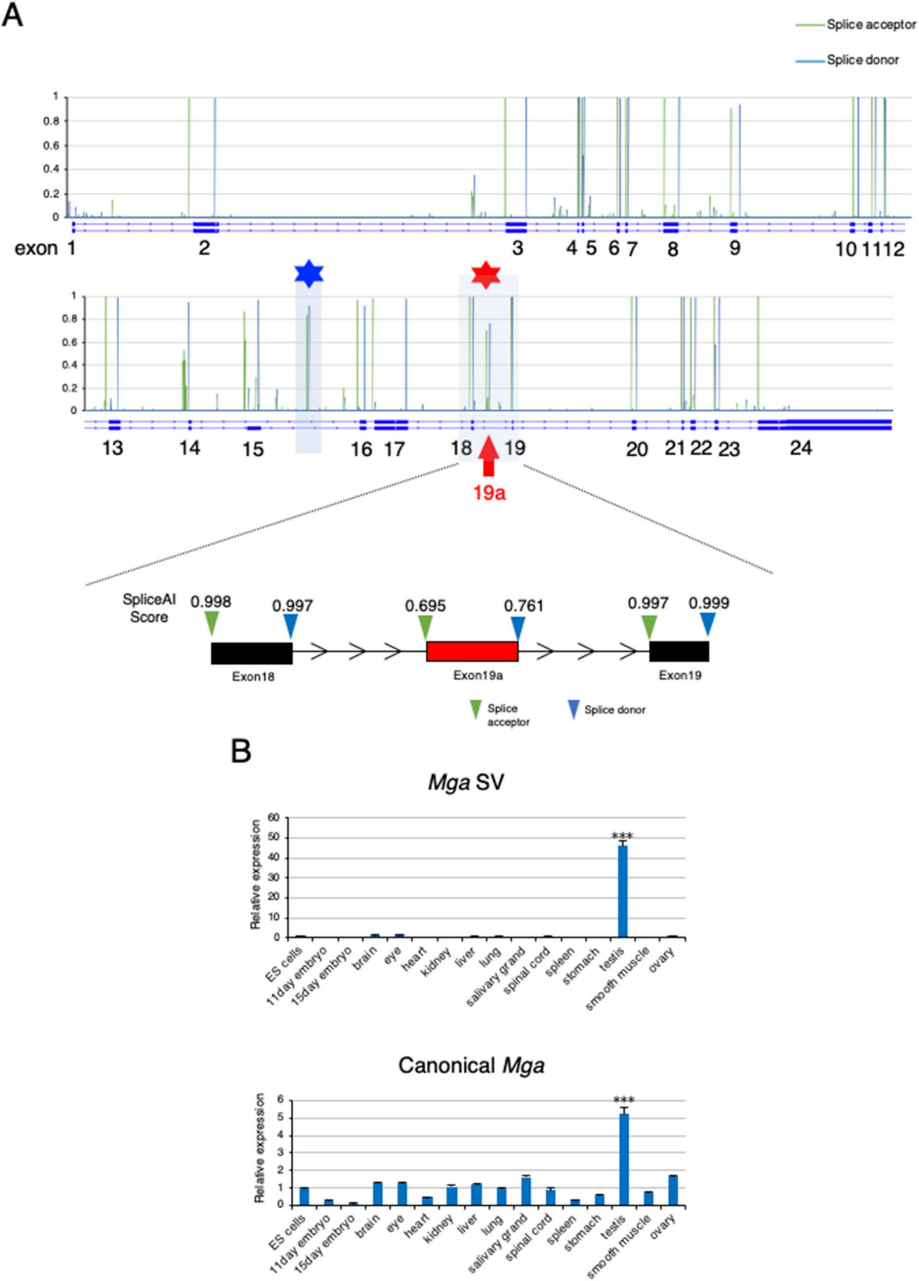
Identification of testis-specific *Mga* variant mRNA. (**A**) Prediction scores as the splice acceptor and donor of *Mga* pre-mRNA from SpliceAI deep learning. Scores as the splice acceptor and donor are shown as green and blue bars, respectively, whose height is proportional to the score level. In addition to known exons, SpliceAI indicated a set of high scores for the splice acceptor and donor within the regions of the 15th and 18th introns of the *Mga* gene marked with blue and red asterisks, respectively. The latter region is enlarged to provide actual scores from SpliceAI. (**B**) qPCR analyses of *Mga* SV (upper panel) and canonical *Mga* (lower panel) mRNAs in total RNAs from various tissues. Values obtained from ESCs were arbitrarily set to one for both *Mga* mRNA species. Data represents the mean ± standard deviation of three independent experiments. The Student’s t-test was conducted to examine statistical significance. ***P < 0.001.

Based on these data, we explored the possibility that the SE type of alternative splicing, particularly gain of a novel exon, was involved in the inactivation of PRC1.6 during meiosis in germ cells. Because SpliceAI, a deep neural network that predicts mRNA splicing from a pre-mRNA sequence^23^, was developed recently, we used this technology to identify putative exon sequences within genes encoding a component of PRC1.6. First, we confirmed that SpliceAI identified all exons of genes encoding a component of PRC1.6 (*Mga*, *Max*, *L3mbtl2*, *E2f6*, *Rnf2*, and *Pcgf6*) (Fig. 1A, Supplementary Fig. S2), which validated the accuracy of prediction by this deep learning program. More importantly, SpliceAI additionally predicted six genomic regions as putative exons (one each within *E2f6* and *Pcgf6* genes and two each in *Mga* and *L3mbtl2* genes) (Fig. 1A, Supplementary Fig. S2). Therefore, we performed RT-PCR analyses of RNAs from various tissues to determine the possibility that RNAs transcribed from these regions were incorporated as exon sequences into mature mRNA in certain tissues (Supplementary Fig. S3). These analyses revealed that a putative exon located within the 18th intron of the *Mga* gene was specifically incorporated into RNA from the testis, but only marginal presence was evident in other tissue RNAs. We also confirmed testis-specific incorporation of the transcript from this region by quantitative PCR (qPCR) (Fig. 1B, upper panel). However, we found that the other five regions were not entirely incorporated into any examined tissue RNAs. According to the scores calculated by SpliceAI (0.695 and 0.761 for the splice acceptor and donor, respectively), this region was not predicted to be a constitutive exon (more than 0.9), but as an exon subjected to a substantial degree of alternative splicing regulation (usually between 0.1 and 0.9). Thus, our finding that this region was used for testis-specific alternative splicing was compatible with the prediction of SpliceAI. Therefore, we termed this sequence and *Mga* variant mRNA carrying this sequence as exon 19a and *Mga* splice variant (SV), respectively. With respect to canonical *Mga* mRNA, we confirmed broad expression in various cell types. We also noted its relatively higher expression in the testis compared with any other examined tissues (Fig. 1B, lower panel).

### RNA in situ hybridization analyses of canonical and variant *Mga* mRNAs in the testis and epididymis

**Figure 2:**
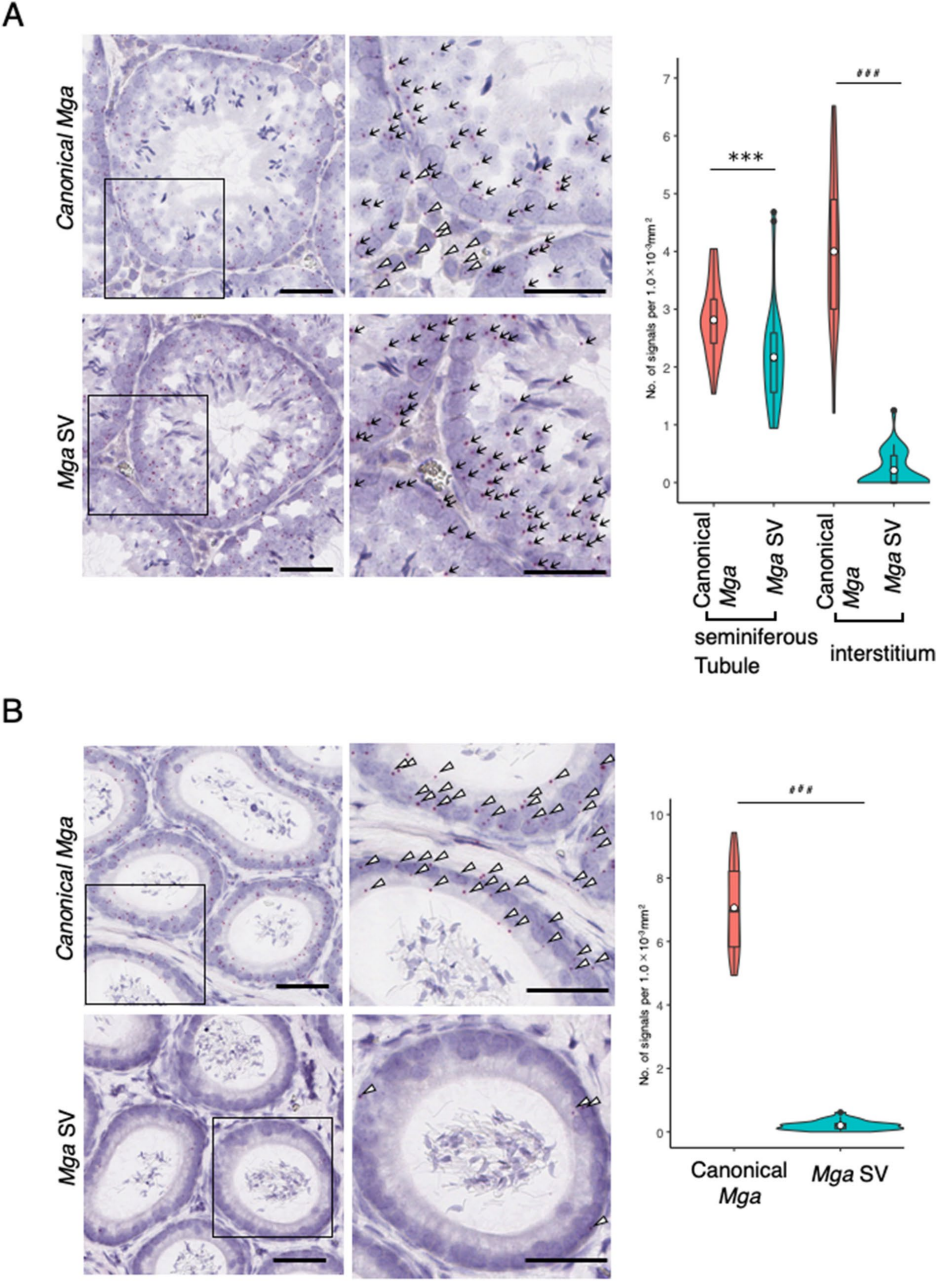
RNA in situ hybridization analyses of canonical and splice variant *Mga* mRNAs in the testis and epididymis. (**A**) Detection of canonical mRNA and its splice variant in adult mouse testis by the BaseScope in situ hybridization. The region indicated with an open square is enlarged and shown at the right. Black arrow and open arrowhead indicate transcripts detected within seminiferous tubule (ST) and interstitium (I) portions, respectively. Black bars correspond to 50 μm. Data obtained as the numbers of positive signals per 1.0 mm^−3^ area in 35 randomly selected areas were used to construct violin plots. For statistical analyses, F-test values were first obtained for the respective data. Then, the Student’s and Welch’s t-tests were conducted when the F-test value was larger and smaller than 0.05, respectively. ***P < 0.001 (Student’s t-test); ^###^*P* < 0.001 (Welch’s t-test) (**B**) Detection of canonical and splice variant *Mga* mRNAs in the epididymis by the BaseScope in situ hybridization. Detection of canonical *Mga* mRNA and its splice variant in the adult mouse epididymis and construction of violin plots as described in A. Black bars correspond to 50 μm. Region indicated with an open square is enlarged and shown at the right. Specific signals for canonical (upper panels) and variant (lower panels) *Mga* mRNAs are indicated with an open arrowhead. Data were analyzed statistically as described in A. ^###^*P* < 0.001 (Welch’s t-test).

To determine which portions of the testis expressed *Mga* SV, RNA in situ hybridization analyses were performed using testis and epididymis tissues of adult mice. To avoid the difficulty associated with the short length (145 bp) of the *Mga* SV-specific sequence, we employed the BaseScope in situ hybridization technique rather than the conventional RNA hybridization method^26^. These analyses clearly demonstrated that *Mga* SV-positive cells were not present in the interstitium portion, but restrictively present within seminiferous tubules, while canonical *Mga*-positive cells were detected in both portions (Fig. 2A). These analyses also revealed that cells positive for canonical *Mga* and those for *Mga* SV were abundantly and scarcely detected in the epididymis, respectively (Fig. 2B).

### *Mga* splice variant is restrictively present around the meiotic stage of germ cells

**Figure 3:**
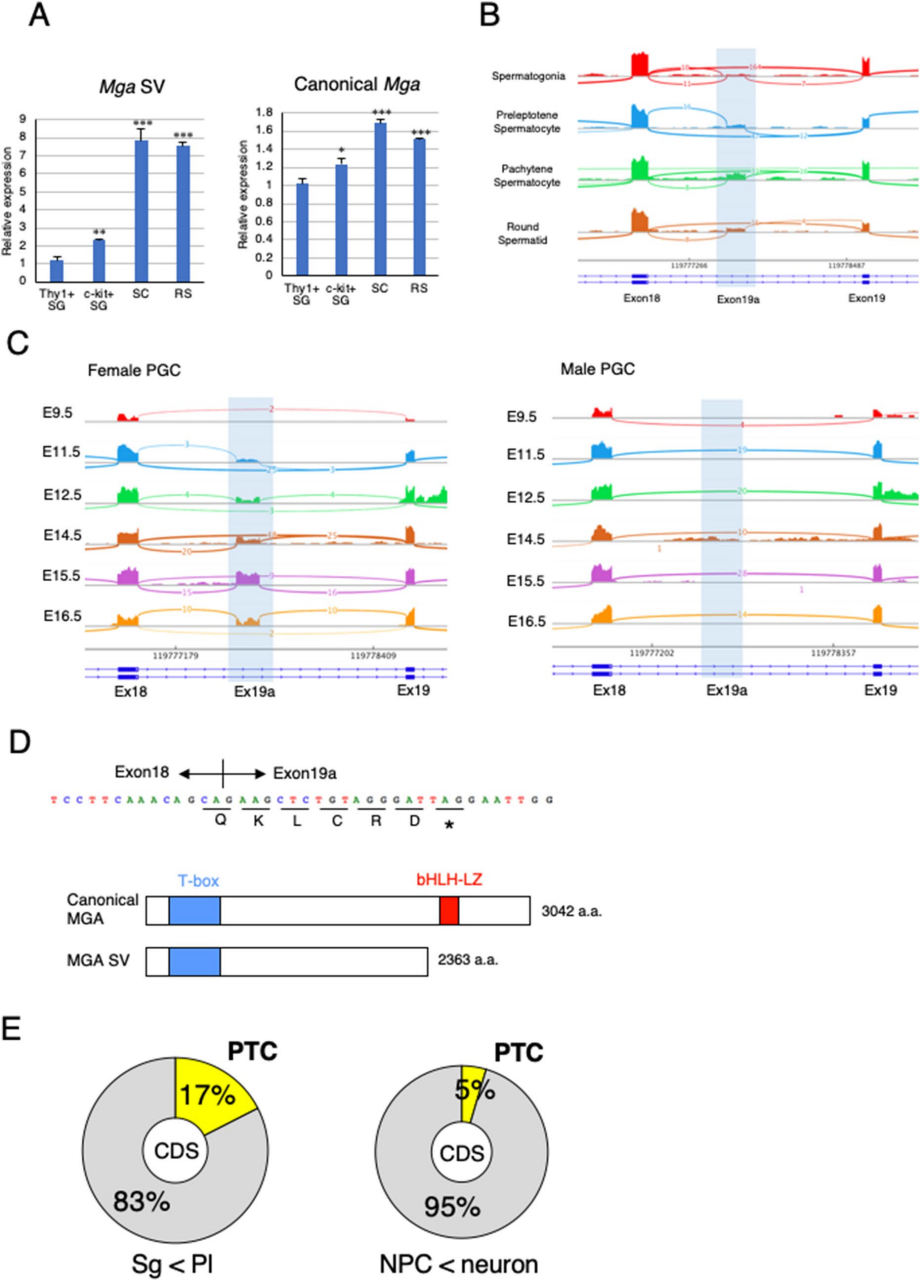
*Mga* splice variant is restrictively present around the meiotic stage of germ cells. (**A**) qPCR analyses of RNAs from undifferentiated (Thy1^+^) and differentiated (c-Kit^+^) spermatogonia (SG), spermatocytes (SC), and round spermatids (RS) to determine the levels of *Mga* SV (left panel) and canonical *Mga* (right panel) mRNAs. Values obtained from Thy1^+^ spermatogonia were arbitrarily set to one for both *Mga* mRNA species. Data were subjected to statistical analyses as described in Fig. 1B. **P < 0.01; ***P < 0.001 (**B**) Visualization of splicing events around exons 18 and 19 of the *Mga* gene during spermatogenesis in the testis by Sashimi plots. Plots were constructed using publicly reported data (GSE75826). Abbreviations are described in (**A**). (**C**) Sashimi plots showing splicing events around exons 18 and 19 of the *Mga* gene in male and female PGCs during the embryonic stage. Data for female and male PGCs were obtained from publicly reported data (E-MTAB-4616) and shown in left and right panels, respectively. (**D**) Sequence of exon 19a containing the in-frame PTC. Acquisition of the exon 19a sequence by alternative splicing was accompanied by insertion of PTC after the addition of the coding sequence of five amino acids, leading to translation into the carboxy-terminally truncated anomalous MGA protein that lacked the bHLHZ domain as depicted in lower portion. (**E**) Generation of the PTC-containing transcript by alternative splicing was a frequent event during meiotic onset of germ cells. Frequencies of exon inclusion leading to incorporation of a PTC and that leading to addition of the novel amino acid sequence occurred during conversion of spermatogonia to meiotic germ cells (left) and differentiation of neural progenitor cells into neuronal cells (right) are presented as pie charts. Publicly reported RNA sequence data of germ cells (GSE75826) and neuronal cells (GSE96950) were used to calculate the frequencies.

To determine whether restrictive expression of *Mga* SV in cells within seminiferous tubules of the testis represented specific expression in germ cells, we prepared four distinct germ cell types, i.e., undifferentiated (Thy1^+^) and differentiated (c-Kit^+^) spermatogonia from testes of mouse pups at postnatal days 5–8 and spermatocytes and round spermatids from adult mouse testes. Then, qPCR analyses were conducted using total RNAs from these cell populations. The analyses revealed that the levels of canonical *Mga* mRNA were comparable among the four cell populations, whereas much higher expression of *Mga* SV was evident in spermatocytes and spermatids compared with Thy1^+^ and c-Kit^+^ spermatogonia in which meiotic initiation is blocked^27^ (Fig. 3A). Next, we examined publicly reported RNA sequence data of this sequence in spermatogonia, round spermatids, and germ cells undergoing meiosis in the testis (preleptotene and pachytene spermatocytes). In accordance with our qPCR data (Fig. 3A), visualization of publicly reported RNA sequence data of this putative exon (exon 19a) by Sashimi plot revealed specific production of *Mga* SV in meiotic spermatocytes and round spermatids, but not in spermatogonia (Fig. 3B). Male germ cells initiate meiosis in the testis at the beginning of puberty, whereas female primordial germ cells (PGCs) undergo meiosis and proceed to the diplotene stage of meiotic prophase I in the gonads during the mid-gestation stage^28,29^. Therefore, we inspected the publicly reported RNA sequence data to determine whether female PGC-specific onset of meiosis in the embryonic stage was also accompanied by the production of *Mga* SV. The analyses revealed that expression of *Mga* SV in female PGCs of the gonads became detectable from 11.5 to 16.5 dpc with peak expression at 15.5 dpc when meiotic germ cells are mostly at the pachytene stage, whereas no apparent peak for the production of *Mga* SV was detected in male germ cells during embryonic stage (Fig. 3C). Taken together, our data demonstrated an intimate link in the time scale between expression of *Mga* SV and meiosis in both male and female germ cells. Because female PGCs undergo meiosis at around 13 dpc, initiation of *Mga* SV production appeared to occur prior to meiotic onset. It is also noteworthy that exon 19a bears in-frame stop codon (Fig. 3D), indicating that the coding sequence located downstream of the stop codon of exon 19a sequence, including that for the bHLHZ domain, was nullified as depicted. Intriguingly, inspection of the publicly reported RNA sequence data revealed that insertion of a PTC in conjunction with gain of a new exon was approximately three times more frequent in the transition from spermatogonia to germ cells at the preleptotene stage compared with differentiation of neural progenitor cells (Fig. 3E). These results indicated that production of *Mga* SV with a PTC represented rather common alternative splicing of new exon inclusion in meiotic germ cells.

### Inefficiency of NMD substantially contributes to accumulation of *Mga* SV transcripts in meiotic spermatocytes and spermatids

**Figure 4:**
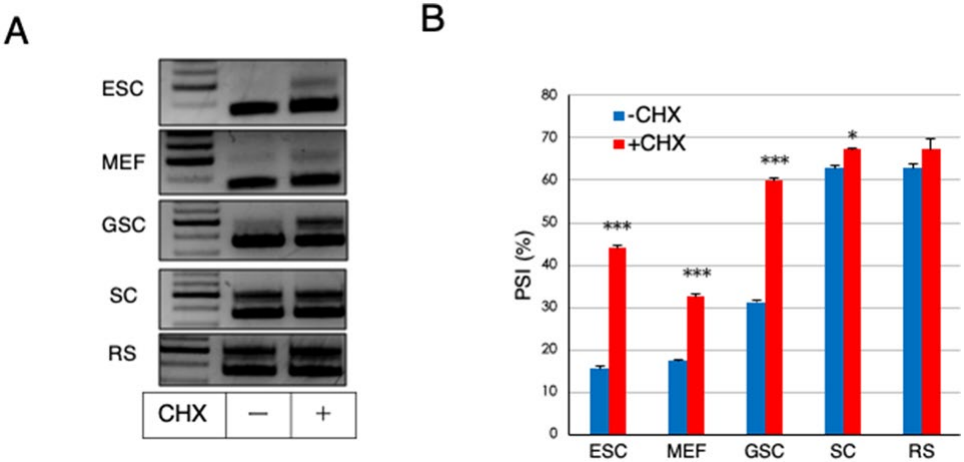
Inefficiency of NMD substantially contributes to accumulation of *Mga* SV transcripts in meiotic spermatocytes and spermatids. (**A**) High expression of *Mga* SV in spermatocytes and round spermatids was not further elevated by suppressing NMD activity. Non-germ (ESC and MEF) and germ (GSC, SC, and RS) cells were treated with or without CHX for 3 h. Semi-quantitative PCR was performed with cDNAs corresponding to their total RNAs. Full-length gels are presented in Supplementary Fig. S7. (**B**) Quantification of canonical and splice variant *Mga* mRNAs in non-germ and germ cells treated with or without CHX. cDNAs used in A were subjected to qPCR to accurately compare their amounts. Data are shown as PSI. Data represent the mean ± standard deviation of three independent experiments. The Student’s t-test was conducted to examine statistical significance. *P < 0.05; ***P < 0.001.

Notably, an in-frame terminating codon was present in exon 19a (Fig. 3D). In general, transcripts containing a PTC are subjected to rapid degradation by NMD^30–32^. However, meiotic and post-meiotic germ cells in the testis are rather defective for PTC-mediated NMD, but not long 3′-untranslated region (UTR)-mediated NMD^33,34^. Therefore, we speculated that an inefficient background of PTC-mediated NMD substantially contributed to the specific presence of the *Mga* SV transcript in meiotic spermatocytes and round spermatids. To test this hypothesis, three germ cell populations [germline stem cells (GSCs), spermatocytes (SCs), and round spermatids (RSs)] and two types of non-germ cells [mouse embryonic fibroblasts (MEFs) and embryonic stem cells (ESCs)] were treated with cycloheximide (CHX) that inhibits NMD. Then, RNAs of these cells were used to quantify expression levels of *Mga* SV by semi-quantitative and quantitative PCRs after conversion to cDNAs (Fig. 4A,B). These analyses revealed that CHX treatment clearly augmented the amounts of *Mga* SV transcripts in ESCs, MEFs, and GSCs compared with untreated cells, which indicated that *Mga* SV mRNA produced in these cells was indeed subjected to degradation by NMD. However, no noticeable alterations in the amounts of *Mga* SV due to treatment with CHX were observed in spermatocytes and round spermatids, which was consistent with the notion of low PTC-mediated NMD activity in these cells. We also noted that the levels of *Mga* SV mRNAs in CHX-treated ESCs and MEFs were significantly lower than those in spermatocytes and round spermatids, whereas CHX-treated GSCs showed almost equivalent levels of *Mga* SV mRNAs as those in spermatocytes and spermatids. Therefore, these results indicated that specific accumulation of *Mga* SV mRNAs in spermatocytes and round spermatids represented the combined consequence of two independent phenomena, i.e., preferential production of *Mga* SV mRNA in germ cells including spermatogonia and attenuated NMD activity against PTC-containing mRNAs in meiotic germ cells.

### Dominant negative effect of carboxy-terminally truncated MGA on PRC1.6

**Figure 5:**
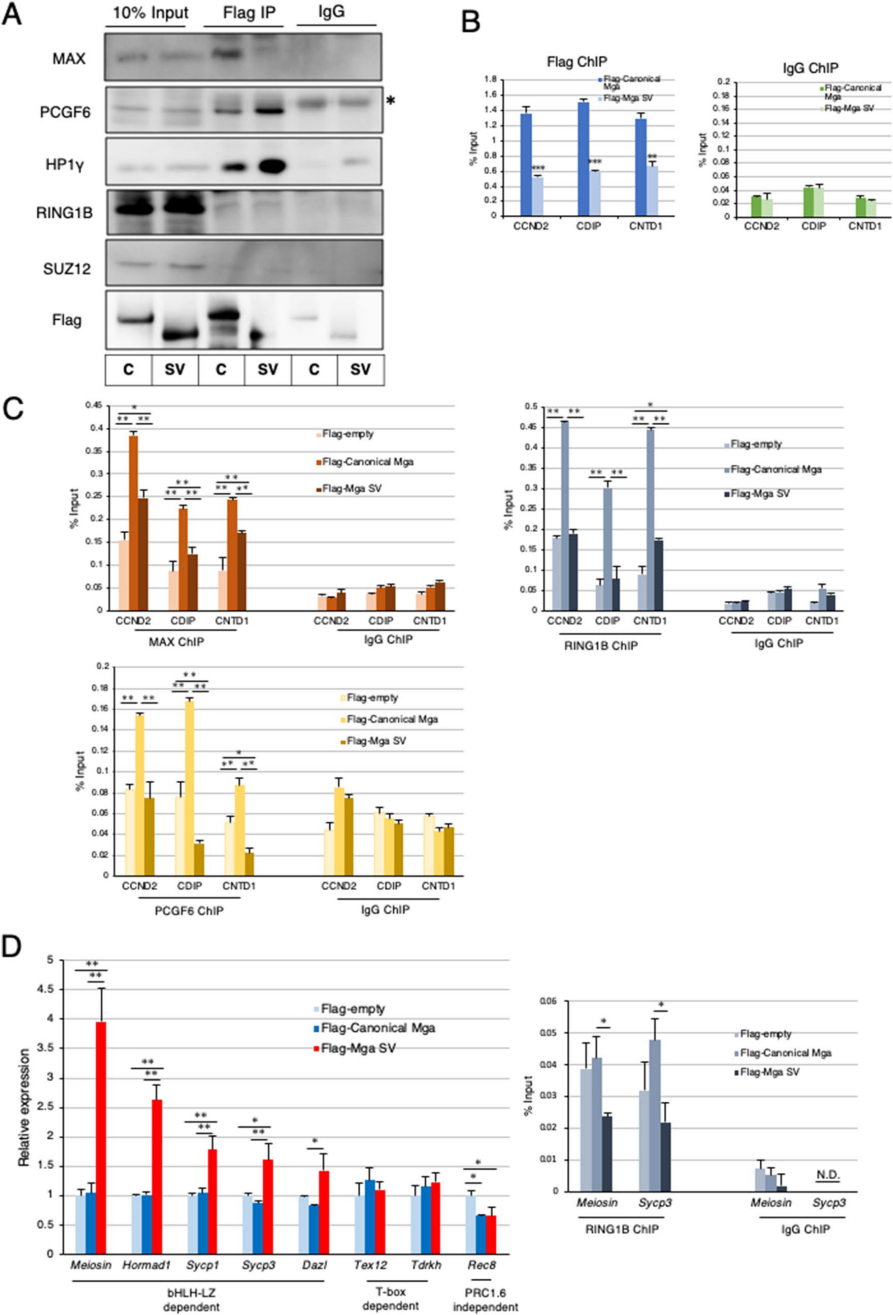
Dominant negative effect of carboxy-terminally truncated MGA on PRC1.6. (**A**) Coimmunoprecipitation analyses of canonical and carboxy-terminally truncated MGAs. Expression vectors for Flag-tagged canonical and carboxy-terminally truncated MGAs were transiently introduced individually into *MGA*-null HEK293FT cells by transfection. Coimmunoprecipatations were performed with an anti-Flag-tag antibody using nuclear extracts from the transfected cells. Coimmunoprecipitated proteins were used to examine the presence or absence of SUZ12 as well as PRC1.6 components (PCGF6, L3MBTL2, HP1γ, and RING1B). C and SV stand for canonical- and splice variant, respectively. *Indicates signals of the immunoglobulin heavy chain used for immunoprecipitation. Full-length blots are presented in Supplementary Fig. S7. (**B**) ChIP-qPCR analyses of PRC1.6-target genes in *MGA*-null HEK293FT cells producing Flag-tagged canonical or carboxy-terminally truncated MGA transiently with the anti-Flag-tag antibody. Control IgG was used as a negative control. Data represent the mean ± standard deviation of three independent experiments. The Student’s t-test was conducted to examine statistical significance. **P < 0.01; ***P < 0.001. (**C**) ChIP-qPCR analyses of PRC1.6-target genes in *MGA*-null HEK293FT cells with empty vector and those producing either Flag-tagged canonical or carboxy-terminally truncated MGA transiently with antibodies against MAX, RING1B or PCGF6. Control IgG was used as a negative control. Data represent the mean ± standard deviation of three independent experiments. The Tukey–Kramer test was conducted to examine statistical significance. *P < 0.05; **P < 0.01. (**D**) Effects of forced expression of *Mga* SV on meiosis-related genes in mouse ESCs Expression vectors to produce Flag-tagged canonical and carboxy-terminally truncated MGAs and that with no cDNA (empty) were transiently introduced individually into mouse ESCs by transfection. Transfected cells were collected as GFP-positive cells. Then, RNAs prepared from them were used to quantify expression levels of meiosis-related genes (left panel). The same sets of ESCs were also used for ChIP-qPCR analyses of *Meiosin* and *Sycp3* promoter loci with antibody against RING1B (right panel). Control IgG was used as a negative control. *N.D.* not detected. Data represent the mean ± standard deviation of three independent experiments. Data were subjected to statistical significance examination as in C. *P < 0.05.

To explore the possible function of MGA SV, we first examined the ability of the protein to interact with other PRC1.6 components by coimmunoprecipitation analyses (Fig. 5A). These analyses revealed no noticeable difference in the efficiency of the interaction with endogenous PCGF6, HP1γ, and RING1B in HEK293FT cells between flag-tagged canonical MGA and its derivative, i.e., MGA SV, which were forcedly produced by transient transfection. Our data also demonstrated that both types of MGA proteins did not bind to SUZ12, a component of PRC2. In addition, we confirmed that canonical MGA, but not MGA SV, interacted with MAX efficiently as expected. Next, we examined the effect of forced production of MGA SV on interactions of PRC1.6 components with genomic DNA. To this end, we also used the HEK293FT cell line. The advantage of using the HEK293FT cell line was complete disruption of *MGA* loci to eliminate the contribution of endogenous MGA in these cells without affecting their viability. In fact, we generated *MGA-*KO HEK293FT cells using the CRISPR-Cas9 system (Supplementary Fig. S4) and used them to examine the interaction of overexpressed canonical and anomalous MGAs with the promoters of PRC1.6 target genes (*CCND2, CDIP*, and *CNTD1*) whose repression is crucially dependent on the bHLHZ domain of MGA in these cells^17^. These analyses revealed that canonical MGA bound much more efficiently to all three gene promoters than MGA SV in *MGA*-null HEK293FT cells (Fig. 5B). At present, we do not know why the interaction scores obtained with MGA SV were not sufficiently low enough to judge as background. However, it is possible that these data represent binding of MGA SV to DNA using its T-box domain and/or other components of PRC1.6., i.e., E2F6/DP1 and L3MBTL2, which can lead to direct and indirect binding of the complex to DNA, respectively. Our analyses also revealed that binding of MAX, RING1B, and PCGF6 to these gene promoters was much less efficient with forced expression of *Mga* SV than that with forcedly produced canonical MGA (Fig. 5C), which further validated that MGA SV is defective in functioning as an active component of PRC1.6. We assume that relatively higher MAX-ChIP-qPCR signal obtained with *MGA*-null HEK293FT cells in which empty vector had been introduced than the signal obtained with control IgG may represent its genomic binding via interaction with other bHLHZ domain-containing proteins such as c-MYC and MAD. Likewise, relatively higher RING1B-ChIP-qPCR signal obtained with control *MGA*-null HEK293FT cells than background level may represent its binding as a component of other PRC1 such as PRC1.1 and PRC1.5.

Next, we used mouse ESCs in which PRC1.6 functions as a blocker of ectopic meiosis^17,20–22^. We transiently introduced expression vectors for canonical *Mga* and *Mga* SV and an empty vector individually in mouse ESCs and then examined alterations in the expression levels of meiosis-related genes. Our analyses revealed that some meiosis-related genes (*Meiosin, Hormad1*, *Sycp1*, *Sycp3*, and *Dazl*) indeed showed significant elevation in their expression levels after overexpression of *Mga* SV, but not canonical *Mga*. However, other PRC1.6 target genes (*Tex12* and *Tdrkh*) did not show appreciable alterations in its expression levels after overexpression of canonical *Mga* or *Mga* SV (Fig. 5D left panel). Because MGA SV lacks the bHLHZ domain, but carries an intact T-box domain, these data were in accordance with our recent observation that repression of *Tex12* and *Tdrkh* genes is dependent on the integrity of the T-box domain of MGA, while the bHLHZ domain of MGA is crucially involved in repressing the expression of *Meiosin, Hormad1*, *Sycp1*, *Sycp3*, and *Dazl* genes^35^. We also found that expression levels of *Rec8* gene, a meiosis-related gene that is not subjected to PRC1.6-dependent regulation, were not elevated, but rather declined by forced expression of *Mga* SV and canonical *Mga*. Consistent with these data, ChIP-qPCR analyses using anti-RING1B antibody revealed that the ChIP-signals at *Meiosin* and *Sycp3* loci of ESCs were significantly declined by forced expression of *Mga* SV, while those were not declined or even elevated by overexpression of canonical *Mga* (Fig. 5D right panel)*.* Next, we examined whether forced *Mga* SV expression further potentiated the magnitude of meiotic induction mediated by *Max* expression ablation. However, these analyses revealed that activated meiosis-related genes in *Max*-null ESCs did not show further activation by overexpression of *Mga* SV (Supplementary Fig. S5), indicating that the effect of production of MGA SV on impediment of PRC1.6 function is much less efficient compared to the effect of *Max* expression ablation. We next investigated physiological alterations in the expression levels of meiosis-related genes during the conversion of spermatogonia to preleptotene spermatocytes in male germ cells using publicly reported RNA sequence data. We found that meiosis-related genes whose repression was crucially dependent on the bHLHZ domain (*Sycp3* and *Sycp1*) profoundly elevated their expression levels during this conversion, whereas those primarily subjected to T-box-dependent repression, such as *Tdrkh* and *Tex12*, were much less significantly activated (Supplementary Fig. S6). These results suggested that de-repression of a subset of meiosis-related genes by forced expression of *Mga* SV in ESCs faithfully recapitulated the physiological activation profile of meiosis-related genes during meiosis.

## Discussion

While the Stra8/Meiosin complex constitutes a positive feedback loop to promote meiotic onset in germ cells^3^, PRC1.6 has the opposite activity^17,20–22^. Therefore, PRC1.6 needs to be inactivated at the timing of meiotic onset to produce a background that allows the Stra8/Meiosin complex to function efficiently and promote meiotic onset. In accordance with this notion, we have previously demonstrated that protein levels of MAX, which constitutes the core of PRC1.6 with MGA, decrease significantly prior to the onset of meiosis in germ cells^21^. In this study, we demonstrated the existence of an additional mechanism to alleviate the repressing activity of PRC1.6. Indeed, we demonstrated that *Mga* splice variant mRNA is specifically present in meiotic spermatocytes and postmeiotic round spermatids, which encodes an MGA protein lacking the bHLHZ domain and thus functions as a dominant negative regulator of PRC1.6.

Thus, we have demonstrated previously^21^ and in this study that at least two molecular mechanisms that target either Max or Mga operate in germ cells independently to impede the functions of PRC1. However, since alternative splicing for *Mga* SV does not occur prior to meiosis, this alternative splicing may only serve as a safeguard mechanism to ensure meiotic progression rather than as a crucial mechanism for meiotic onset. It is also noteworthy that overlap between germ cells with reduced MAX protein levels and those positive for *Mga* SV mRNA is evident only up to the zygotene stage of meiotic prophase I in germ cells. Indeed, MAX protein levels increased significantly around the pachytene stage, while *Mga* SV mRNA was present during all stages of meiotic cell division and its presence was persistent at least up to the round spermatid stage. Thus, inactivation of PRC1.6 after the zygotene stage may be solely dependent on the dominant negative function of MGA SV generated from translation of *Mga* SV mRNA, although we do not eliminate the possibility that other unknown molecular mechanisms impede PRC1.6 in parallel.

MGA has two distinct DNA-binding domains termed T-box and bHLHZ domains that are independent and dependent on the interaction with MAX to bind to DNA, respectively^17,36,37^. However, it was recently demonstrated by an unbiased de novo sequence motif search that the T-box motif was not identified as a binding motif of PRC1.6 in ESCs^17^. This contrasts with the identification of E-box sequence that is recognized by the MGA/MAX complex via their bHLHZ domains as a highly enriched binding motif for PRC1.6. Moreover, our recent study demonstrated that deletion of bHLHZ domain of MGA in ESCs is accompanied by derepression of numerous meiosis-related genes that play pivotal roles in meiotic progression, such as *Meiosin* and *Sycp3*. However, only a few genes that have been demonstrated to be crucially involved in meiosis are significantly activated by deletion of the T-box domain^35^. Although these data were obtained from studies using ESCs that bear the potential for ectopic meiosis, these studies strongly suggest that the bHLHZ domain has a much more predominant role in suppressing meiosis than the T-box domain. Therefore, we consider that production of the dominant negative MGA protein that lacks the bHLHZ domain via alternative splicing is a reasonable strategy for germ cells to promote meiosis. Consistent with this assumption, we also noted that meiosis-related genes whose repression is strongly dependent on bHLH-LZ, such as *Sycp1* and *Sycp3*, dramatically elevated their expression levels during physiological conversion of spermatogonia to preleptotene spermatocytes, whereas those primarily subjected to T-box-dependent repression, such as *Tdrkh* and *Tex12*, were not profoundly activated.

Unusual mRNAs such as those with a PTC including *Mga* SV are usually degraded rapidly by NMD^38^. However, it is known that PTC-dependent, but not long 3′-UTR-dependent, NMD, is defective in spermatocytes and round spermatids. Indeed, it has been demonstrated that conditional knockout of the *Upf2* gene, which is essential for NMD, in these cells is not associated with further accumulation of PTC-containing transcripts^33,39^. As the molecular mechanisms underlying this weak NMD activity in spermatocytes and round spermatids, *Upf3a* and *Upf3b* genes involved in the repression and activation of NMD, respectively, are transcriptionally up- and down-regulated in these germ cells. Moreover, it has been demonstrated that conditional knockout of *Upf3a* in *Stra8*-positive meiotic germ cells is accompanied by substantial reductions in the levels of PTC-containing mRNAs in spermatocytes^40^. We hope our future in vivo studies certify that production of anomalous MGA is one of representative examples of exquisite utilization of the defective background of PTC-dependent NMD in meiotic spermatocytes and round spermatids for an obvious biological phenomenon.

In summary, our computational search identified a potential exon within the *Mga* gene and our subsequent analyses revealed that the sequence was incorporated into canonical mRNA specifically in meiotic germ cells and round spermatids by alternative splicing. Moreover, our data indicate that anomalous MGA protein produced in germ cells may alleviate the repressing activity of PRC1.6 to ensure meiosis by functioning as a dominant negative regulator by exquisitely using the inefficient background of PTC-dependent NMD in these cells.

## Methods

### Approval for mouse experimentation

Six- to eight-week old C57BL/6 mice were inbred at the Saitama Medical University animal facility. Animal experimentation was carried out in compliance with the ARRIVE guidelines (http://www.nc3rs.org.uk/page.asp?id=1357) as well as institutional guidelines of Saitama Medical University. The protocol was approved by the Institutional Review Board on the Ethics of Animal Experiments of Saitama Medical University (permission numbers 2579 and 3032).

### Cell culture

EBRTcH3^41^ and their derivative, *Max*-null^21^ ESCs were cultured in Glasgow minimum essential medium (SIGMA-ALDRICH) containing 10% fetal bovine serum and leukemia inhibitory factor (1000 U/ml). Mouse GSCs (kindly provided by Dr. Takashi Shinohara, Kyoto University, Japan) were cultured on mitomycin C-treated MEFs as described by Kanatsu-Shinohara et al.^42^. HEK293FT cells were cultured in Dulbecco’s modified Eagle’s medium containing 10% fetal bovine serum.

### Isolation of germ cells

To isolate Thy1^+^ and c-Kit^+^ spermatogonia, testes from C57BL/6 mouse pups at postnatal days 5–8 were collected and then minced after removal of the tunica albuginea membrane. After two consecutive enzymatic digestions using collagenase type I and trypsin along with DNase I, cells were recovered as floating cells in gelatin-coated tissue culture plates to enrich the germ cell population. The recovered cells were then subjected to magnetic activated cell sorting to isolate CD117 (c-Kit)^+^ cells followed by isolation of CD90 (Thy1)^+^ cells using the same method.

Spermatocytes and round spermatids were isolated from testes of 8–12-week-old adult C57BL/6 mice. After conducting the procedure described above, the testicular cell suspension was subjected to Percoll density gradient centrifugation and spermatocyte- and round spermatid-enriched fractions were isolated as fractions with 30% and 22% Percoll densities, respectively, by inspecting their nuclear morphology with an aid of Hoechst staining.

### RT-PCR and qPCR analyses

TRIzol Reagent (THERMO FISHER SCIENTIFIC) was used for total RNA preparation from sorted ESCs by flow cytometry. Total RNAs from other samples and cDNAs were prepared as described previously^21^. RT-PCR was performed using TaKaRa Ex Taq Hot Start Version or PrimeSTAR Max DNA Polymerase (TAKARA). PCR products were subjected to agarose gel electrophoresis. TaqMan and SYBR Green-based qPCRs were performed using the StepOnePlus Real-Time PCR System (APPLIED BIOSYSTEMS). To identify *Mga* variant mRNA by qPCR, one of the primers was set within a putative exon sequence (exon 19a). All samples were tested in triplicate and the results were normalized to *Gapdh* expression levels. Primer sequences and TaqMan probes are listed in Supplementary Table S1.

### In situ hybridization for canonical and splice variant *Mga* mRNAs

Formalin-fixed, paraffin-embedded sections of the testis and epididymis of adult ICR mice were used for BaseScope in situ hybridization assays employing a BaseScope Red reagent kit (ADVANCED CELL DIAGNOSTICS)^26^ to detect canonical and splice variant *Mga* mRNAs. The sections were counterstained with hematoxylin.

### Cycloheximide treatment

ESCs, MEFs, and GSCs were treated with CHX (100 μg/ml) for 3 h. Spermatocytes and spermatids isolated from 8-week-old C57BL/6 mice were suspended in αMEM with 10% knockout serum replacement and 10 μg/ml GDNF (PEPROTECH # 450-10), and then treated with or without CHX (100 μg/ml) for 3 h.

### Expression vector construction

To construct expression vectors for canonical MGA and MGA SV, corresponding cDNAs recovered by PCR were individually introduced into the pCAG-IRES-Puro eukaryotic expression vector^43^ using in-fusion technology after subcloning oligonucleotides carrying initiating methionine codon followed by a Flag-tag sequence. pCAG-IRES-EGFP eukaryotic expression vector was constructed by replacing Puro cDNA with EGFP and used for the experiments shown in Fig. 5D.

### Generation of homozygous *MGA*-knockout HEK293FT cells

The CRISPR/Cas9 system was used to establish *MGA*-null HEK293FT cells. Oligonucleotide pairs carrying the same sequence used by Stielow et al.^17^ were inserted into the pX330-U6-Chimeric_BB-CBh-hSpCas9 vector. The vector was then introduced into HEK293FT cells together with pCAG-mycGFP-IN by cotransfection using Lipofectamine 2000 (THERMO FISHER SCIENTIFIC). Subsequently, GFP-positive cells were collected by fluorescence-activated cell sorting and seeded a 96-well tissue culture plate with one cell per each well. Genomic DNAs prepared from individual cell colonies were used to identify clones in which the *MGA* gene was homozygously disrupted.

### Nuclear extract preparation, and coimmunoprecipitation and western blot analyses

Nuclei were prepared from 3 × 10^7^ HEK293FT cells by lysing the cell membrane under hypotonic conditions followed by low speed centrifugation (1000 rpm). Then, nuclei were resuspended in hypertonic buffer containing 300 mM NaCl and placed on ice for 15 min. A nuclear extract was prepared as the supernatant after moderately high speed centrifugation (10,000 rpm) as described by Uranishi et al.^44^. For coimmunoprecipitation analyses, an anti-Flag antibody or normal mouse IgG were added to the nuclear extract together with Dynabeads bearing anti-Mouse IgG (THERMO FISHER SCIENTIFIC). After incubation for 1 h with gradual rotation at 4 °C, proteins bound to beads were eluted with sample buffer after extensive washing with PBS containing 0.1% BSA, heated, and then resolved by SDS-PAGE. Separated proteins were electrophoretically transferred to PVDF membranes. The membranes were blocked in PBST containing 5% dry skim milk and subjected to western blot analyses with primary antibodies followed by appropriate secondary antibodies conjugated with horseradish peroxidase. Antibodies used are listed in Supplementary Table S2.

### ChIP-qPCR analyses

For ChIP-qPCR analyses with *MGA*-null HEK293FT cells, suspended cells were fixed with 1% paraformaldehyde in PBS for 10 min at room temperature and then washed extensively with PBS. The cells were resuspended in cell lysis buffer (10 mM Tris–HCl, pH 8.1, 10 mM NaCl, 1.5 mM MgCl_2_, and 0.5% Igepal-CA630) with a protease inhibitor cocktail and placed on ice for 15 min. After centrifugation, pellets were resuspended in nuclear lysis buffer (50 mM Tris–HCl, pH 8.1, 5 mM EDTA, and 1% SDS) with a protease inhibitor cocktail and then sonicated. After centrifugation, the supernatant was subjected to an immunoreaction with a specific antibody pre-conjugated with magnetic protein A beads (MILLIPORE) overnight at 4 °C. The beads were washed twice with TE buffer after consecutive washes with low salt wash buffer (0.1% SDS, 1% Triton X-100, 2 mM EDTA, 20 mM Tris–HCl, pH 8.1, and 150 mM NaCl), high salt wash buffer (0.1% SDS, 1% Triton X-100, 2 mM EDTA, 20 mM Tris–HCl, pH 8.1, and 500 mM NaCl), and LiCl wash buffer (1% Igepal-CA630, 1% deoxycholate, 1 mM EDTA, 10 mM Tris–HCl, pH 8.1, and 250 mM LiCl). Crosslinks were cleaved by heat treatment. Genomic DNAs associated with beads were recovered using a QIAquick PCR Purification Kit (QIAGEN) and then used to quantify their amounts by qPCR. For ChIP-qPCR analyses with ESCs, empty vector of pCAG-3× Flag-IRES-EGFP and that with Mga *SV* were individually introduced in ESCs by transfection. After 48 h post transfection, 3 × 10^5^ GFP-positive cells were collected as transfected cells by fluorescence-activated cell sorting and were used for ChIP-qPCR analyses using ChIP-IT High Sensitivity kit (ACTIV MOTIF) according to the manufacturer.

### Publicly reported RNA sequencing data analyses

To compare alterations in the frequency and predominant types of alternative splicing during spermatogenesis, RNA sequence data reported by Lin et al.^45^ (GSE75826) were retrieved. Likewise, to assess the dynamics of alternative splicing during conversion of neural progenitor cells to neuronal cells and differentiation of MSCs into osteoblasts, RNA sequence data reported by Liu et al.^46^ (GSE96950) and Shao et al.^47^ (GSE112694) were retrieved, respectively. To construct the Sashimi plots shown in Fig. 3B,C, RNA sequence data reported by Lin et al.^45^ (GSE75826) and Sangrith et al.^48^ (E-MTAB-4616) were used, respectively. Retrieved fastq files were mapped to mouse genome version mm9 with HISAT2 version 2.1.0 and analyzed using the Tuxedo protocol^49^. The resulting SAM files were converted to BAM files using Samtools version 1.9 and sorted by read name^50^.

### Alternative splicing analyses

Aligned bam files were analyzed using MISO version 0.5.4. The threshold to be judged as differentially expressed between two different cell types was set to 10 in Bays factor. Index of “percentage spliced in (PSI)” that denotes the efficiency of inclusion of a specific exon into the transcript population of a gene was calculated according to Katz et al.^51^. ∆PSI represents PSI value of a sample subtracted from that of other (or control) sample. Sashimi plots of RNA sequence reads were generated by Integrative Genomics Viewer^52^.

## Supplementary Information

Supplementary Information.

## Data Availability

All data generated or analyzed during this study are included in this published article or its Supplementary information files.
