# Supplementary material for: Identification of germ cell-specific Mga variant mRNA that promotes meiosis via impediment of a non-canonical PRC1
Source: Sci Rep. 2021 May 6;11:9737. doi: 10.1038/s41598-021-89123-5 (PMC8102552; doi:10.1038/s41598-021-89123-5)
Supplement: Supplementary file 1 — Supplementary Information. [file 41598_2021_89123_MOESM1_ESM.pdf]

Supplementary Information

Identification of germ cell-specific *Mga* variant mRNA that promotes meiosis via impediment of a non-canonical PRC1

Yuka Kitamura, Kousuke Uranishi, Masataka Hirasaki, Masazumi Nishimoto, Ayumu Suzuki, Akihiko Okuda

This file contains seven supplementary Figures and two supplementary Tables.

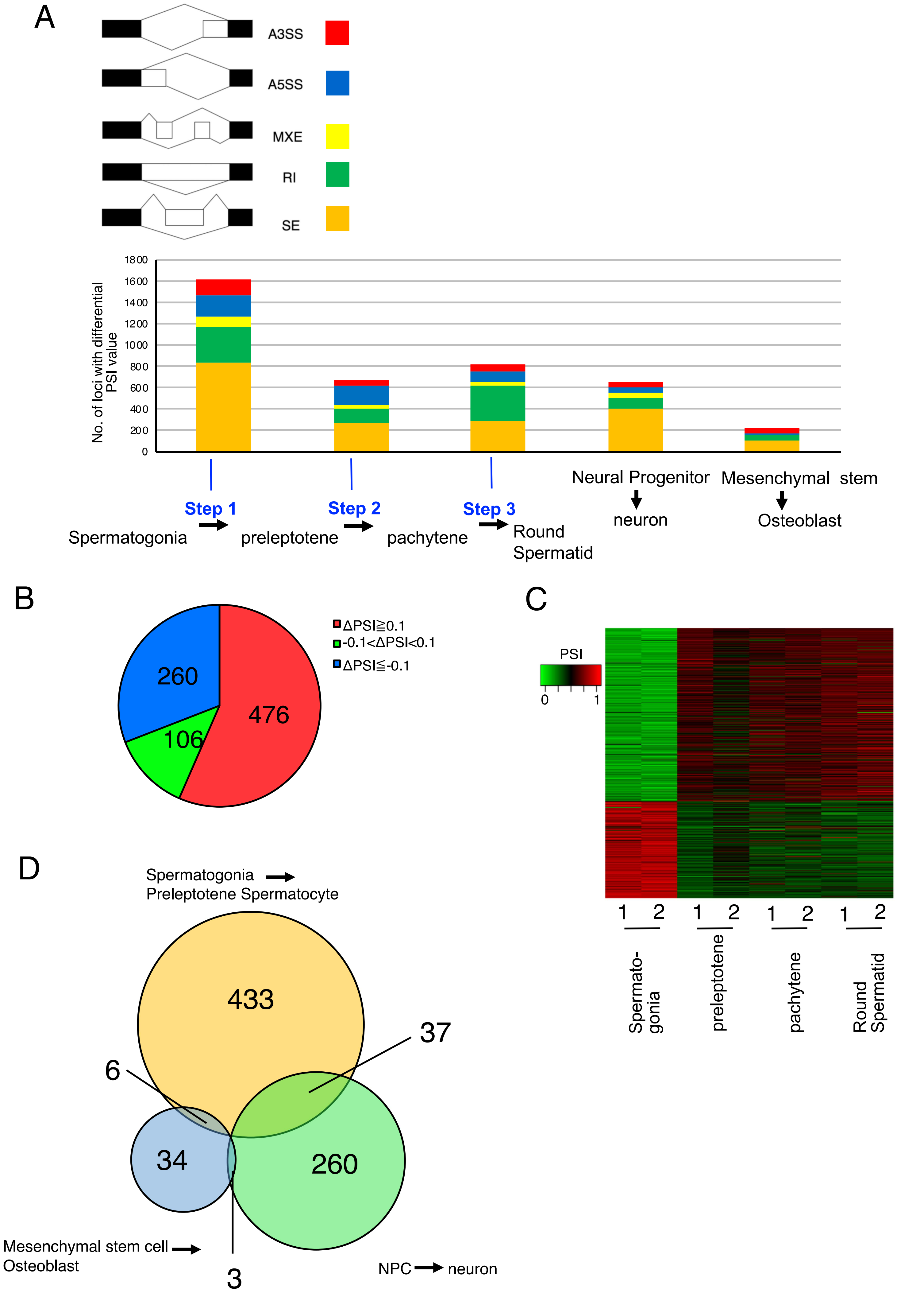

Supplementary Figure 1

Supplementary Figure 1. SE type is the most predominant alternative splicing during meiotic onset in germ cells (A) Comparisons of frequency and the types of alternative splicing among the three different transitions in stage of spermatogenesis. Five different types of alternative splicing are schematically shown at the top. Publicly reported RNA sequence data were used to obtain the PSI in the four different germ cell types indicated in the schema. A bar graph was constructed after calculating changes in the PSI (ΔPSI) at steps 1–3 individually. Data from differentiations of neural progenitor cells and mesenchymal stem cells are also shown as references. A3SS: alternative 3' splice site; A5SS: alternative 5' splice site; MXE: mutually exclusive exon; RI: retained intron; SE: skipping exon (B) Classification of SE type alternative splicing into three subgroups according to the range of ΔPSI. (C) Transcripts with an increased or decreased PSI during meiotic onset were maintained at least up to round spermatids. Genes with PSIs that were significantly increased or decreased at the stage corresponding to step 1 in A were selected and their PSIs in spermatogonia, preleptene spermatocytes, pachytene spermatocytes, and round spermatids were plotted. Data were retrieved from two independent experiments (1 and 2) conducted by Lin et al.[45]. (D) Venn diagram showing comparisons of genes with ΔPSIs that is equal or larger than 0.1 in the differentiation of spermatogonia, neural progenitor, or mesenchymal stem cells. No genes that showed 0.1 or larger ΔPSI values upon differentiation were shared among the three different cell types.

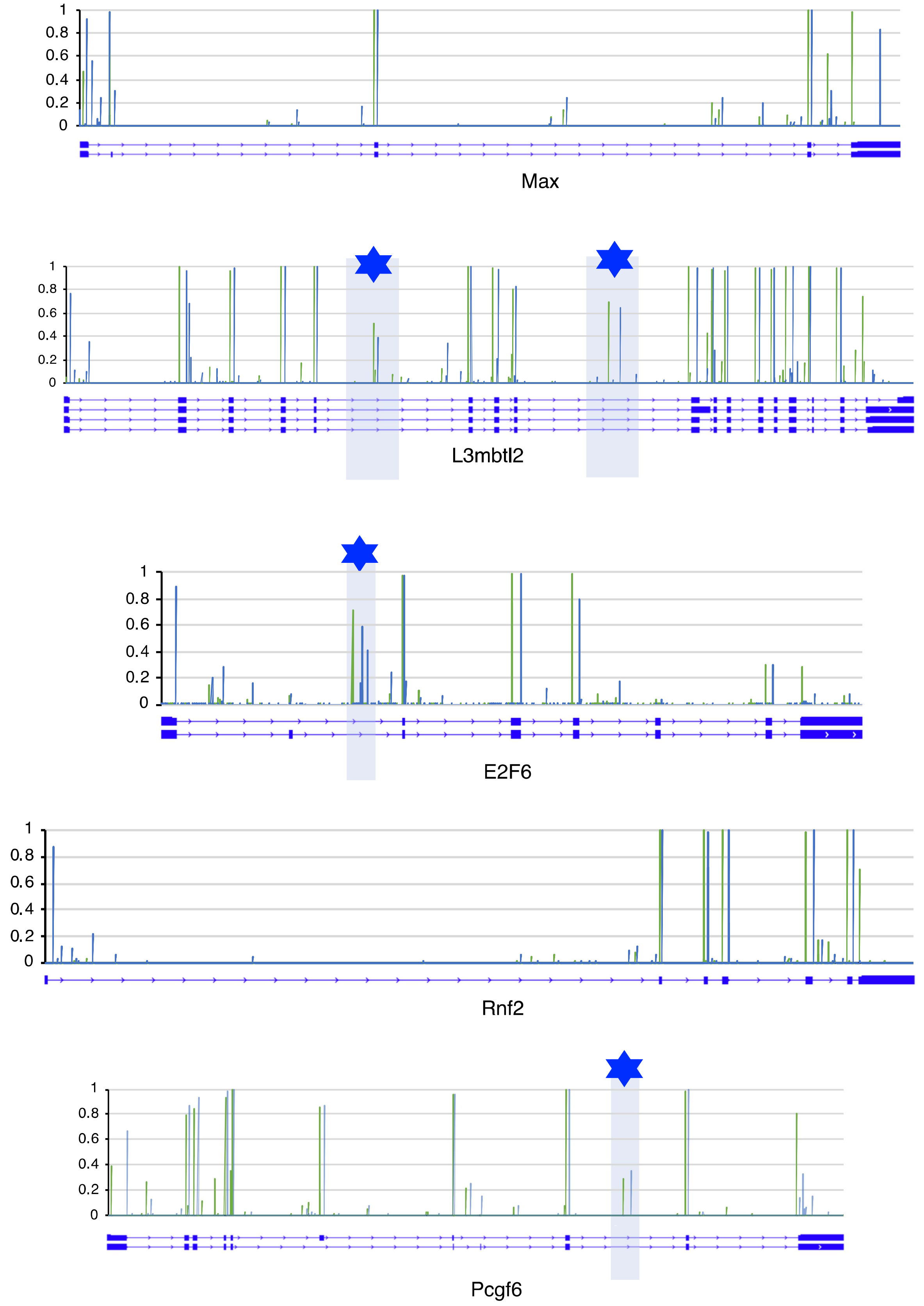

Supplementary Figure 2. Search for potential exons within genes encoding a PRC1.6 component by SpliceAI deep learning. Sequences from pre-mRNA transcripts of genes encoding a PRC1.6 component (*Max, L3mbtl2, E2f6, Rnf2*, and *Pcgf6*) were subjected to the analyses of SpliceAI deep learning. Scores as the splice acceptor and donor are shown as green and blue bars, respectively. Blue asterisks indicate regions with a set of high scores for the splice acceptor and donor determined by SpliceAI except for known exons.

Supplementary Figure 2

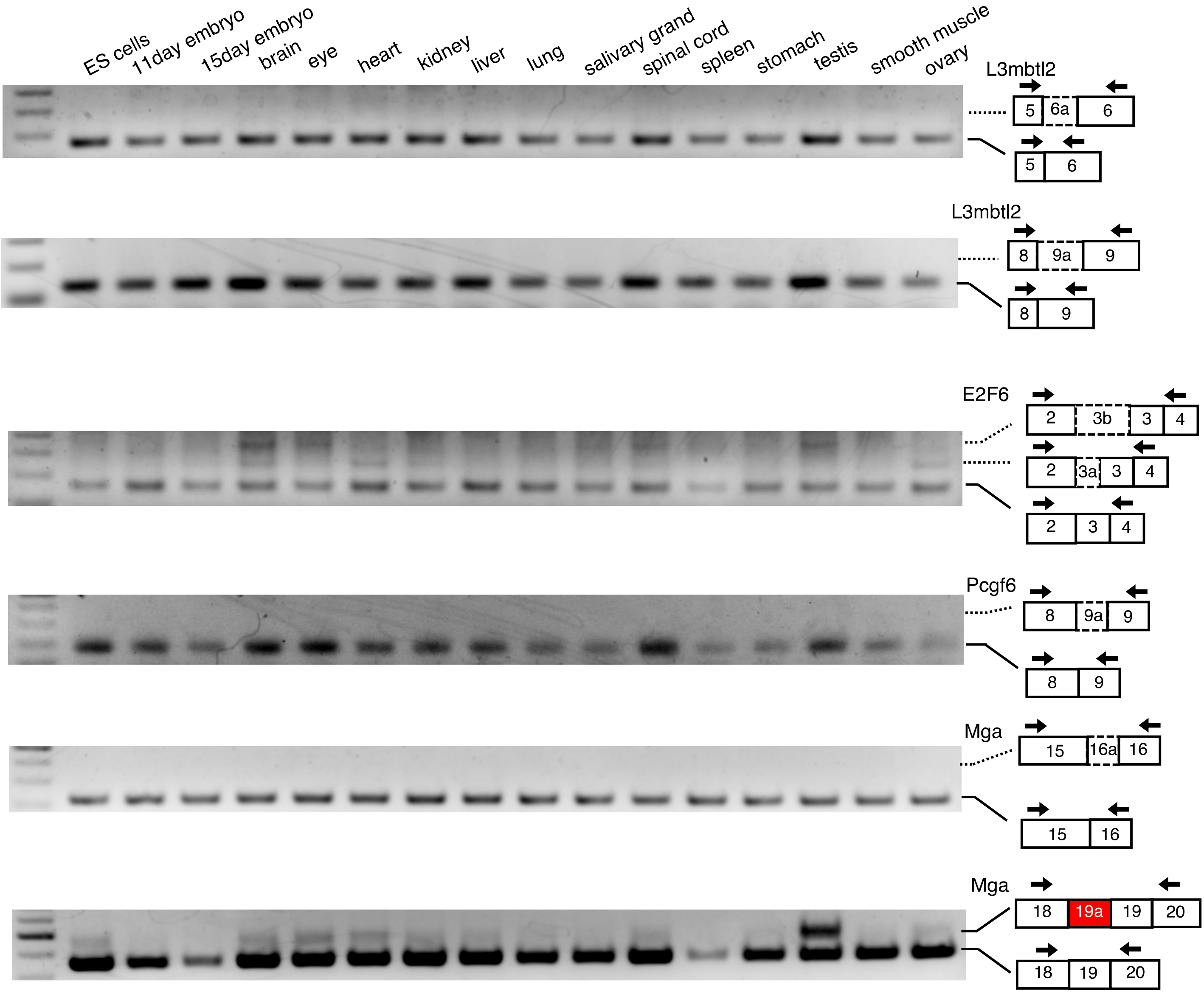

Supplementary Figure 3. RT-PCR analyses of the regions identified as putative exons by SpliceAI. RT-PCR analyses were conducted using 16 different mRNAs with respect to the regions suggested as putative exons by SpliceAI. Several faint and/or smear bands obtained by analyses of the *E2f6* gene in addition to the band corresponding to the canonical mRNA were found to be irrelevant bands by sequencing PCR products. The uncropped full-length gels are presented in Supplementary Figure 7.

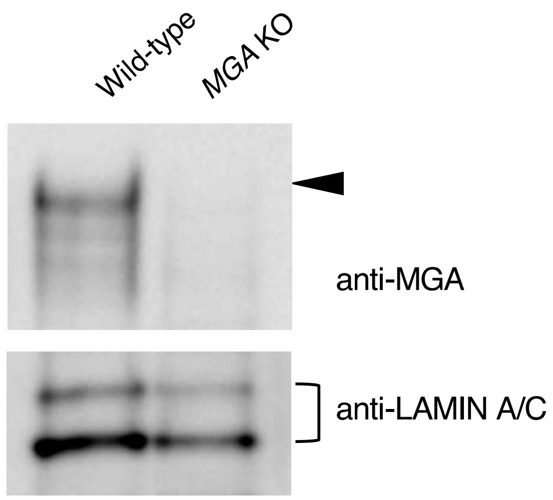

Supplementary Figure 4. Confirmation of the lack of MGA in *MGA*-knockout HEK293FT cells by western blot analyses. MGA and internal control LAMIN A/C proteins were detected by western blot analyses of nuclear extracts from wildtype and *MGA*-null HEK293FT cells. Homozygous knockout of the *MGA* gene in HEK293FT cells was conducted by CRISPR-Cas9-mediated genomic manipulation targeting the region around the 3′-end of exon 3 of the *MGA* gene using oligonucleotide sequences described by Stielow et al.[17]. Generated *MGA*-null HEF293FT cells were identical to those generated by Stielow et al.[17] at the single nucleotide sequencing level, i.e., 73 and 55 bp deletion in the 3′-end of exon 3 and 5′-end of intron 3, respectively, causing abnormal splicing and a frameshift. The nuclear protein-transferred PVDF membrane was cut into two pieces in which upper and lower portions of the filter were used for detecting MGA and LAMIN A/C, respectively, as shown in Supplementary Figure 7.

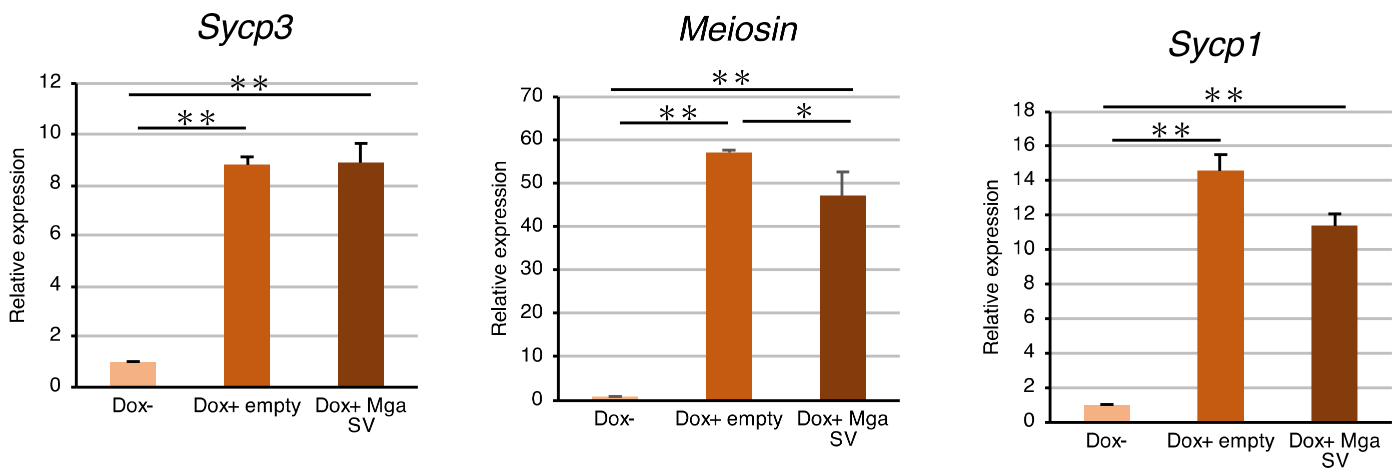

Supplementary Figure 5. Further activation of meiosis-related genes in *Max* expression-ablated ESCs by forced expression of *Mga* SV. Expression levels of meiosis-related genes (*Sycp3*, *Meiosin* and *Sycp1*) were compared between *Max* expression-ablated ESCs in which empty vector had been introduced and those subjected to forced expression of *Mga* SV. *Max* expression was eliminated by treating *Max*-null ESCs with Doxycycline at the concentration of 1 μg/ml as described previously[21]. Doxycycline-untreated *Max*-null ESCs with exogenous *Max* expression from *Rosa26* locus were used as a control. Data represents the mean ± standard deviation of three independent experiments. The Tukey-Kramer test was conducted to examine statistical significance. *P<0.05; **P<0.01

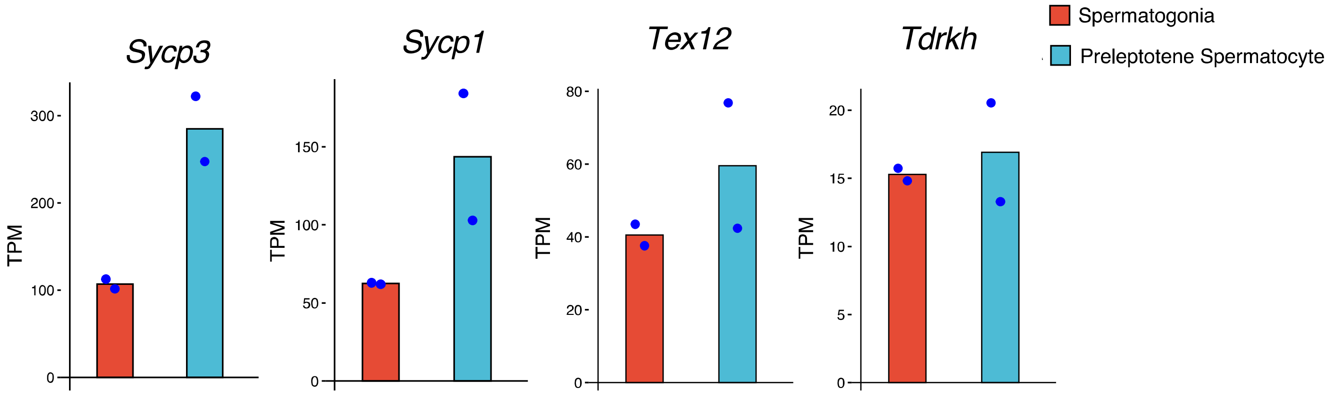

Supplementary Figure 6. Expression levels of meiosis-related genes during meiotic onset in publicly reported RNA sequence data. Expression data of meiosis-related genes that are primarily subjected to regulation by bHLHZ (*Sycp3* and *Sycp1*) or the T-box domain (*Tex12* and *Tdrkh*) of MGA in spermatogonia and preleptotene spermatocytes were extracted from publicly reported RNA sequence data by Lin et al.[45] and shown as a bar graph. Data are shown as the mean of two independent experiments in which each dot represents the value from an individual report.

Supplementary Figure 6

Fig.4A

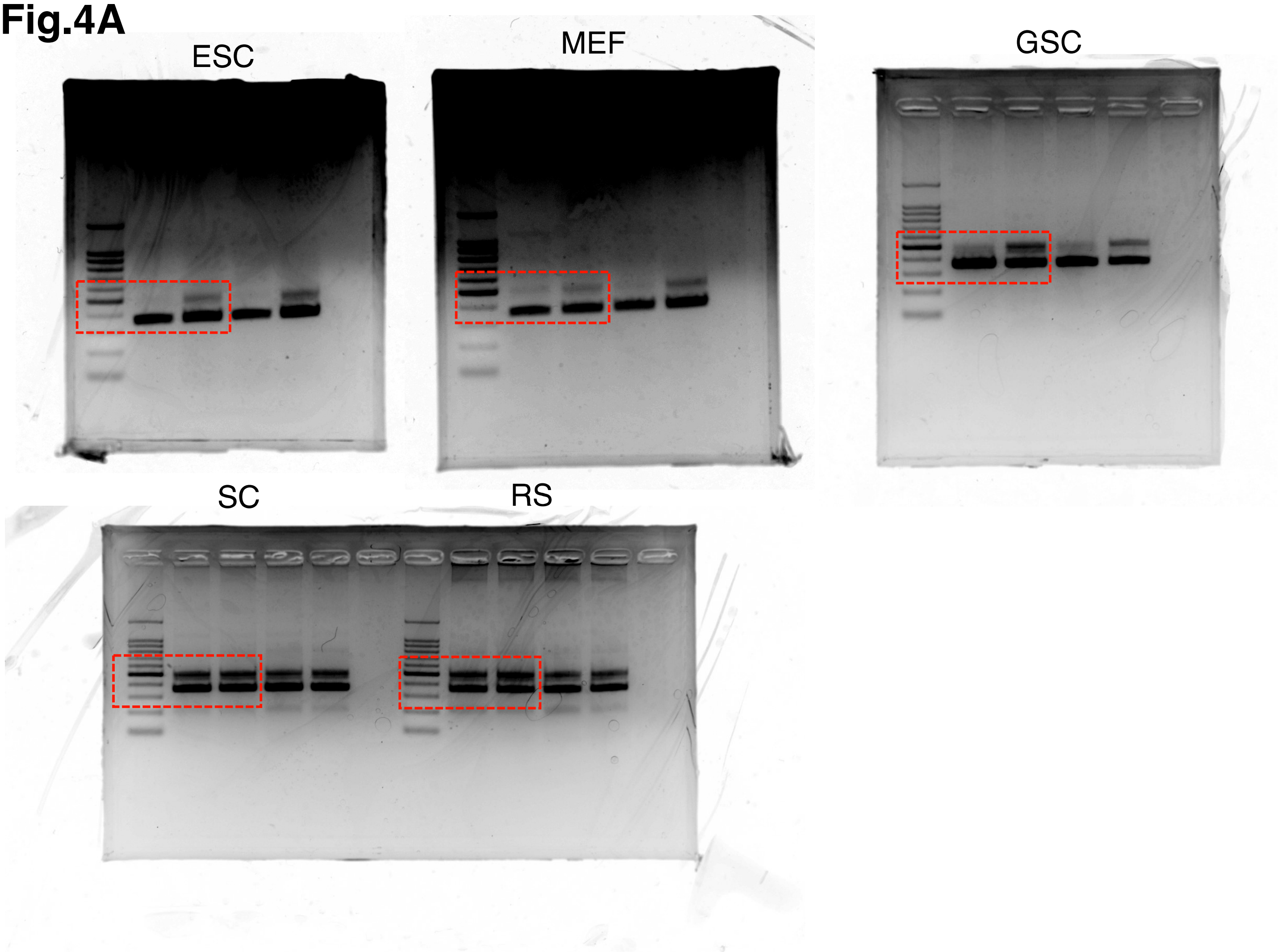

Fig.5A

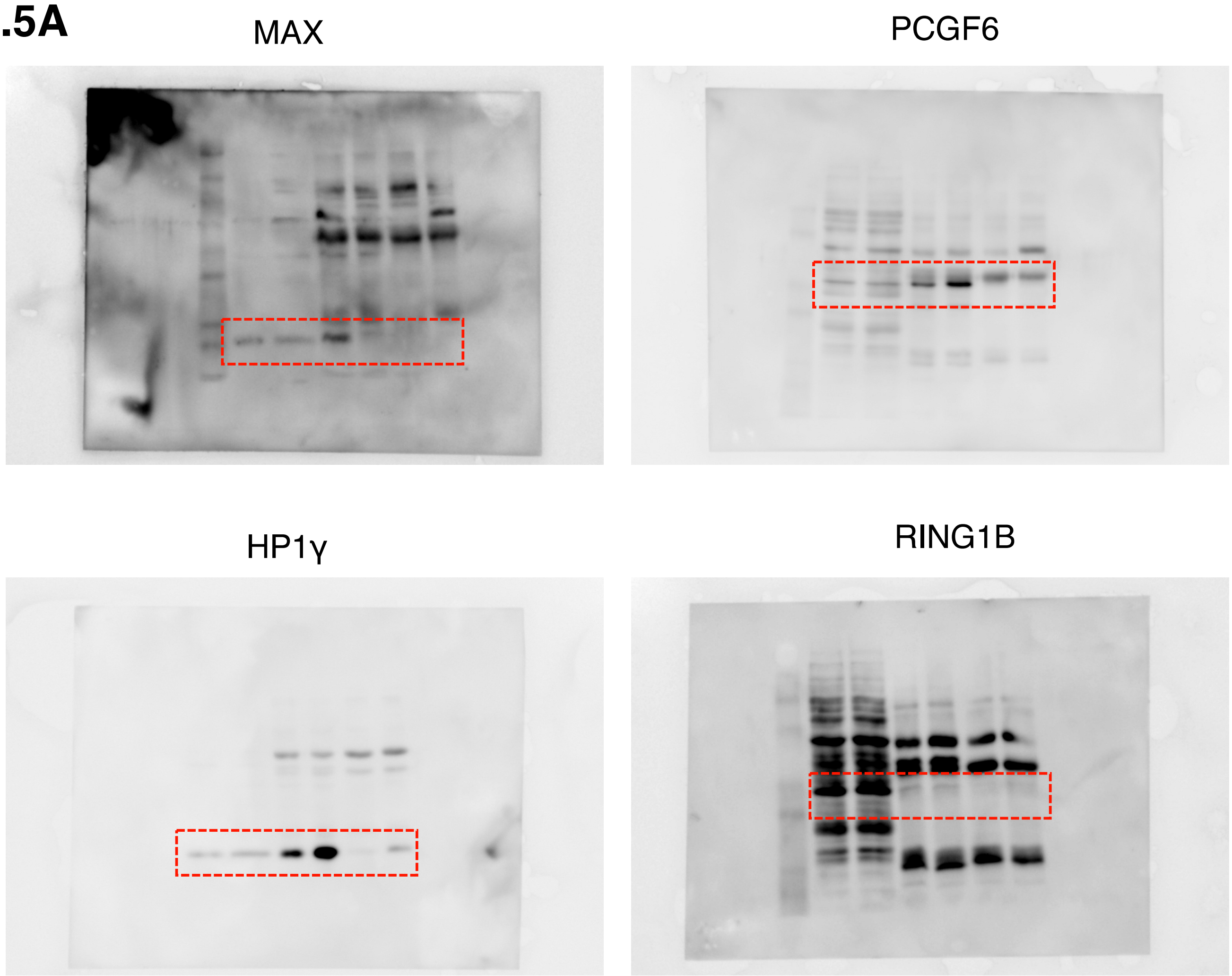

SUZ12

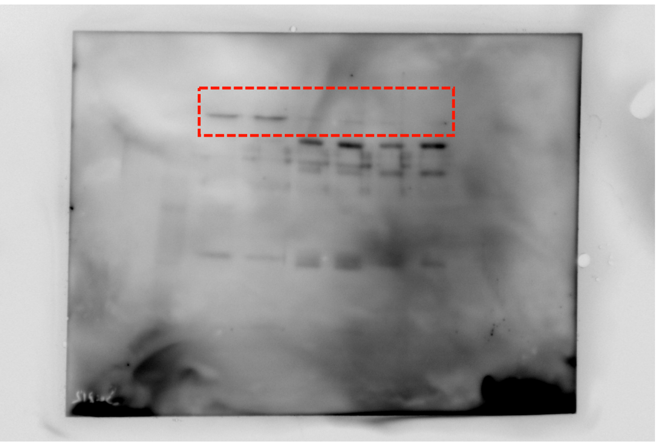

Flag

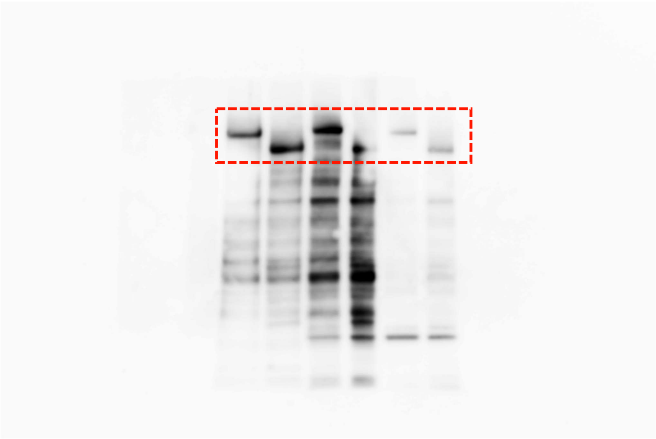

Fig.S3

L3mbtl2 Ex5-6

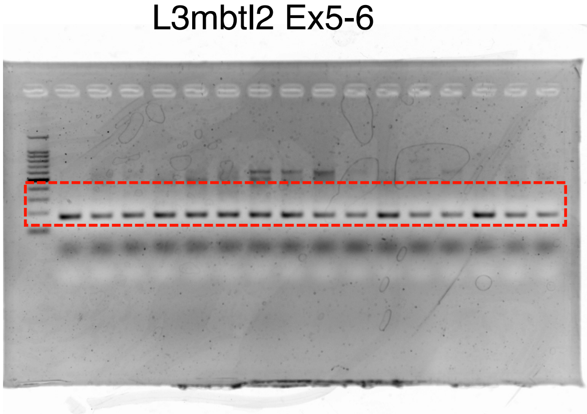

L3mbtl2 Ex8-9

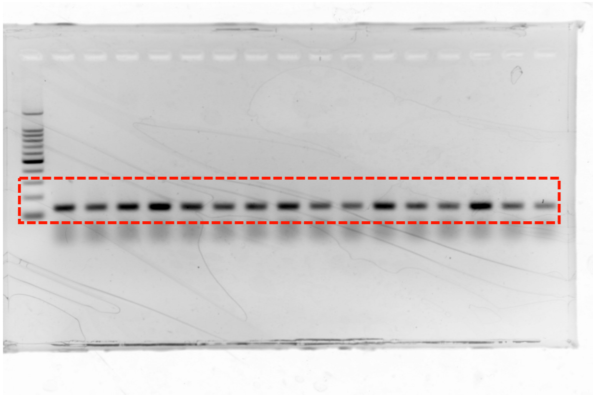

E2F6 Ex2-4

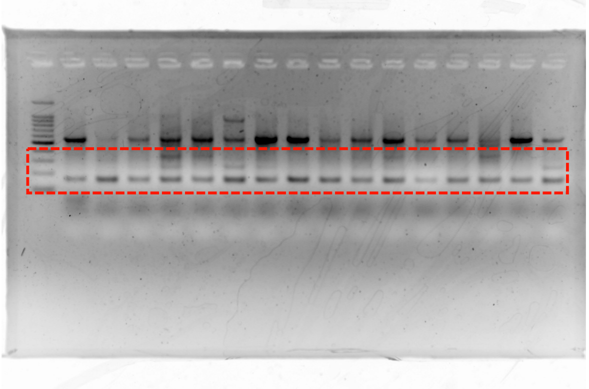

Pcgf6 Ex8-9

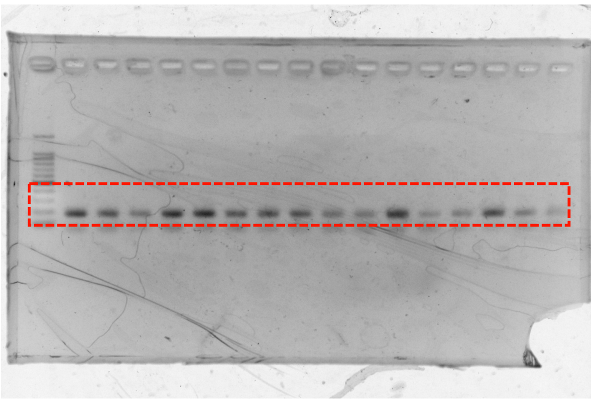

Mga Ex15-16

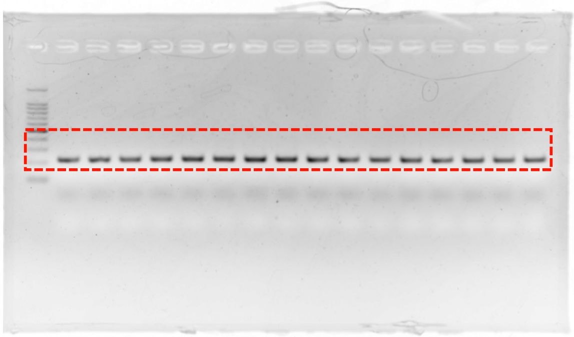

Mga Ex18-19

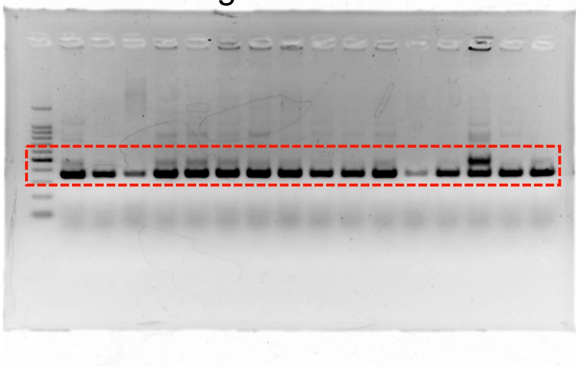

Fig.S4

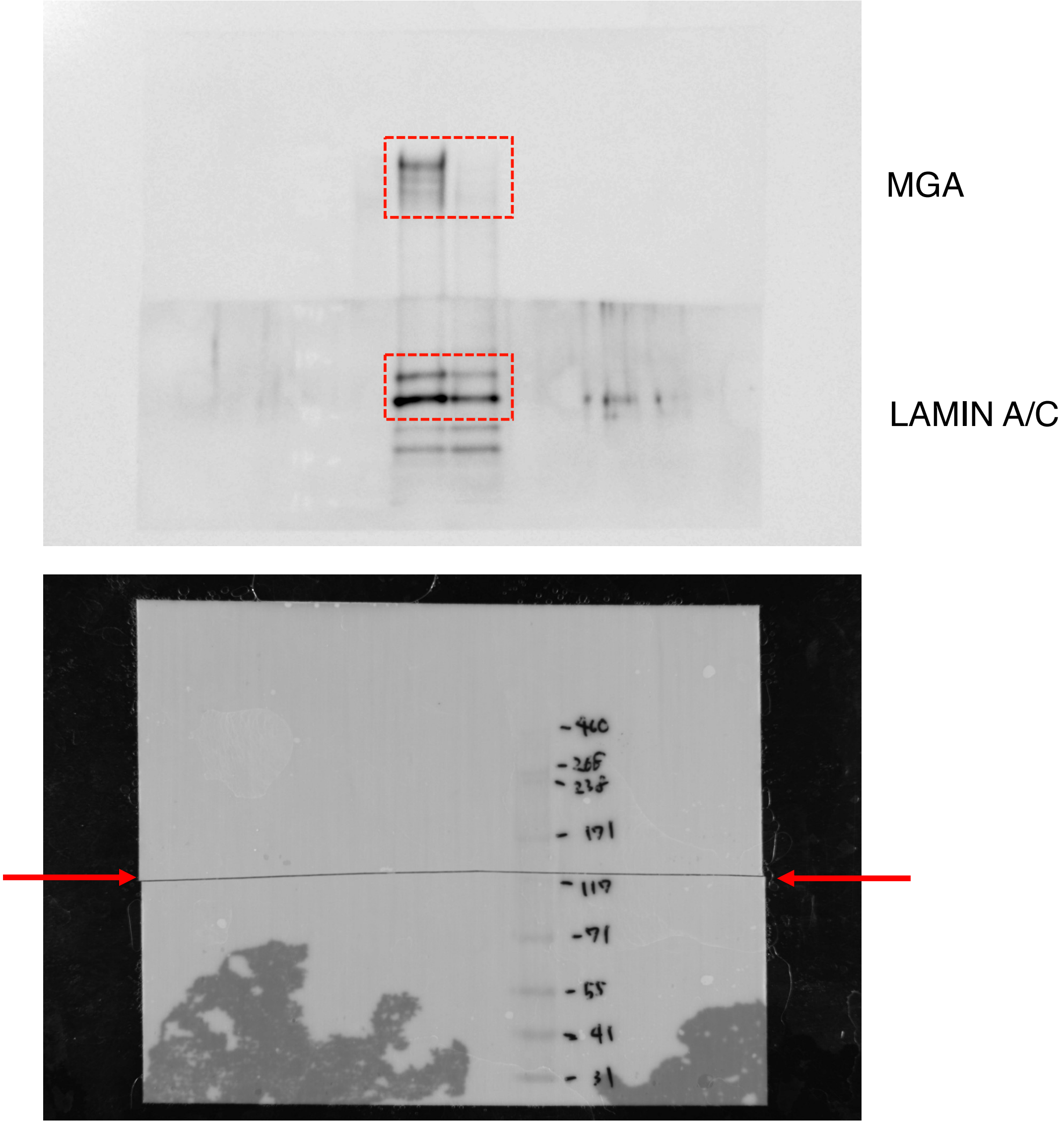

Supplementary Table 1

Oligonucleotides and TaqMan Probes

Oligonucleotides

RT-PCR

L3mbtl2 Exon5-6*	forward	5'-ACTGGGGCAAGTTCCTGAAG-3'
	reverse	5'-TGCCTGGATGACAGTGGCGAT-3'
L3mbtl2 Exon8-9	forward	5'-GAAGAGCTACCTCATGAAGCGG-3'
	reverse	5'-CACCTGAGTCTTGTCTACAACCTC-3'
E2F6 Exon 2-4	forward	5'-CTTCTAGCCAGGTGTGGTGG-3'
	reverse	5'-TACCAGTGACACATCAAACCGG-3'
Pcgf6 Exon8-9	forward	5'-CCATTGGAAAAGAAGTTTGTGCGTG-3'
	reverse	5'-GCTGTATCACCTATTGCACGTCG-3'
Mga Exon 15-16	forward	5'-GGTGACCACACCTACTTCATCACTG -3'
	reverse	5'-TGTCCCTGAAGCTGTGGGTT-3'
Mga Exon18-19	forward	5'-GAGGATGAGGAAGATGAGAAAACTGA-3'
	reverse	5'-TGTCCGTCGGTAATATGCAA-3'

ChIP-qPCR

Human CCND2	forward	5'-CGCCACCAGATCGTATCTCCTGTAA-3'
	reverse	5'-CCTCACTCGCCAGGCTTTCT -3'
Human CDIP	forward	5'-CAGCCTCGTGTACATTGGGCA-3'
	reverse	5'-GAGGCGATTTGGCCTAGAGCT-3'
Human CNTD1	forward	5'-GTAGGACCTTCTGCCACTGGG-3'
	reverse	5'-GAGCTGGTGACCCTCTGGATTCT-3'

qPCR

Meiosin	forward	5'-CATTGACATGACCAAGGCCTTGC-3'
	reverse	5'-TGGAGGGAGTGGAGTGTTGCT -3'
Tex12	forward	5'-GAGAAGGATTTGAGCGATATGAGCAAGG-3'
	reverse	5'-CTGTAAACCTCTGCTTCAGGAACTCTC -3'
Tdrkh	forward	5'-TTCTGGTGCCCAGAGCAGTC-3'
	reverse	5'-GGCTGCGGGAACCAATGATTTG-3'
Gapdh	forward	5'-CTCAATGACAACTTTGTCAAGCTCA-3'
	reverse	5'-TTACTCCTTGGAGGCCATGTAG-3'

CRISPR/Cas9

Human MGA Exon 3	forward	5’-CACCGCATCTGGAAAGGTACTCCCA-3’
	reverse	5’-AAACTGGGAGTACCTTTCCAGATGC-3’
Human MGA intron 3	forward	5’-CACCG TCATACTTGAATTGTATAC-3’
	reverse	5’-AAACGTATACAATTCAAGTATGAC-3’

Genotyping for MGA-KO HEK293FT

Human MGA Exon3-intron3	forward	5’-GAAAGAGCCTCAGTGGAAATATCCTG-3’
	reverse	5’-ATGAAAATTCCAGTAAGACCCGAAGAC-3’

TaqMan probes used for qPCR

**Gene Symbol**	**Probe ID**
Sycp1	Mm01298009_m1
Sycp3	Mm00488519_m1
Hormad1	Mm00471448_m1
Dazl	Mm03053726_s1
Rec8	Mm00490939_m1
Gapdh	Mm99999915_g1
canonical *Mga*	**Custom-made**
	forward 5’-GAAGACCACAGCAACTCACACAC-3’
	reverse 5’-TTTTTCATCTGCAGAGATATGGCTA-3’
	probe 5’-TCCTTCAAACAGCAGTGTC-3’
variant *Mga*	**Custom-made**
	forward 5’-GATTCCTGAGACAGTTTCCTAAGTGA -3’
	reverse 5’-TTTTTCATCTGCAGAGATATGGCTA-3’
	probe 5’-TTCAGTTACCTATTAAGGTGTC-3’

*Gene symbols represent mouse genes if not indicated otherwise.

Supplementary Table 2
Antibodies

Primary Antibodies

Antigen	Manufacturer	Catalog No.	Usage	Remark
mouse MGA	ABCAM	ab214814	WB	rabbit monoclonal
human MGA*			WB	rabbit polyclonal
mouse MAX	SANTA CRUZ	sc-197	ChIP	rabbit polyclonal
human MAX	PROTEINTECH	10426-1-AP 1	WB	rabbit polyclonal
human PCGF6	ABCAM	ab192395	WB	rabbit polyclonal, cross-reacts with mouse PCGF6
mouse PCGF6	ABCAM	ab200038	ChIP	rabbit monoclonal
human HP1γ	SANTA CRUZ	sc-398562	WB	mouse monoclonal, cross-reacts with mouse HP1γ
human RING1B	CELL SIGNALING	#5694	ChIP	rabbit monoclonal, cross-reacts with mouse RING1B
human RING1B	ABCAM	ab101273	WB	rabbit polyclonal, cross-reacts with mouse RING1B
human SUZ12	ABCAM	ab12073	WB	rabbit polyclonal, cross-reacts with mouse SUZ12
human LAMIN A/C	SANTA CRUZ	sc-20681	WB	rabbit polyclonal, cross-reacts with mouse LAMIN A/C
FLAG-tag	SIGMA-ALDRICH	F3165	WB, ChIP, IP	mouse monoclonal
Normal Rabbit IgG	CELL SIGNALING	#2729	ChIP	use as control IgG in ChIP experiments
Normal Mouse IgG1	CELL SIGNALING	#5415	ChIP, IP	use as control IgG in ChIP and IP experiments

Horseradish Peroxidase- Conjugated Secondary Antibodies

Antigen	Manufacturer	Catalog No.	Usage	Remark
rabbit IgG	CELL SIGNALING	#7074	WB	goat polyclonal
mouse IgG	CELL SIGNALING	#7076	WB	horse polyclonal
rabbit IgG	ROCKLAND	18-8816-33	WB	mouse monoclonal
mouse IgG	ROCKLAND	18-8817-33	WB	rat monoclonal

*An antibody kindly provided by Dr. Bastian Stielow at Institute of Molecular Biology and Tumor Research, Philipps-University of Marburg in Germany who had used in their study (PLoS Genet 14, e1007193, 2018)
